# Tyrosinase Degradation Is Prevented when EDEM1 Lacks the Intrinsically Disordered Region

**DOI:** 10.1371/journal.pone.0042998

**Published:** 2012-08-08

**Authors:** Marioara B. Marin, Simona Ghenea, Laurentiu N. Spiridon, Gabriela N. Chiritoiu, Andrei-Jose Petrescu, Stefana-Maria Petrescu

**Affiliations:** 1 Department of Molecular Cell Biology, Institute of Biochemistry of Romanian Academy, Bucharest, Romania; 2 Department of Bioinformatics and Structural Biochemistry, Institute of Biochemistry of Romanian Academy, Bucharest, Romania; Drexel University College of Medicine, United States of America

## Abstract

EDEM1 is a mannosidase-like protein that recruits misfolded glycoproteins from the calnexin/calreticulin folding cycle to downstream endoplasmic reticulum associated degradation (ERAD) pathway. Here, we investigate the role of EDEM1 in the processing of tyrosinase, a tumour antigen overexpressed in melanoma cells. First, we analyzed and modeled EDEM1 major domains. The homology model raised on the crystal structures of human and Saccharomyces cerevisiae ER class I α1,2-mannosidases reveals that the major mannosidase domain located between aminoacids 121–598 fits with high accuracy. We have further identified an N-terminal region located between aminoacids 40–119, predicted to be intrinsically disordered (ID) and susceptible to adopt multiple conformations, hence facilitating protein-protein interactions. To investigate these two domains we have constructed an EDEM1 deletion mutant lacking the ID region and a triple mutant disrupting the glycan-binding domain and analyzed their association with tyrosinase. Tyrosinase is a glycoprotein partly degraded endogenously by ERAD and the ubiquitin proteasomal system. We found that the degradation of wild type and misfolded tyrosinase was enhanced when EDEM1 was overexpressed. Glycosylated and non-glycosylated mutants co-immunoprecipitated with EDEM1 even in the absence of its intact mannosidase-like domain, but not when the ID region was deleted. In contrast, calnexin and SEL 1L associated with the deletion mutant. Our data suggest that the ID region identified in the N-terminal end of EDEM1 is involved in the binding of glycosylated and non-glycosylated misfolded proteins. Accelerating tyrosinase degradation by EDEM1 overexpression may lead to an efficient antigen presentation and enhanced elimination of melanoma cells.

## Introduction

Secretory and membrane proteins are synthesized on bound ribosomes and co-translationally translocated in the endoplasmic reticulum (ER) lumen where folding occurs. The correct folding of a polypeptide is a significant process for its biological function during which the nascent chain adopts a native three dimensional conformation. To cope with the increased wave of newly synthesized proteins, the ER quality control mechanism discriminates between native and incorrectly folded protein [1]. Proteins that do not reach the native conformation are extracted from the ER and destroyed by proteolysis in the cytosol by the ubiquitin-proteasome system. In Eukaryotes there are several mechanisms to eliminate misfolded proteins that could aggregate and impede normal ER functions that are collectively termed ER-associated degradation (ERAD) pathways.

Most of the polypeptides receive precursor N-linked glycans to glycosylation sites located within the amino acid backbone. N-glycans processing and polypeptide folding proceed concomitantly, glycans being involved in the selection of ER-resident lectin-like chaperones and redox proteins that assist protein folding [2]. Sequential trimming of the N-glycans by ER glucosidases generates monoglucosylated glycans (GlcMan9) that are recognized by calnexin/calreticulin. These lectins impede the premature export of the nascent polypeptide chain from the ER [3]. By recognizing the monoglucosylated glycans, calnexin/calreticulin introduces the glycosylated polypeptide into a cycle where de- and re-glucosylation of the glycans are determined by the detection of exposed hydrophobic patches in the presence of the two key proteins, glucosyl transferase and glucosidase II [4]. After undergoing several cycles, correctly folded proteins are released from the cycle and exported from the ER. Incorrectly folded proteins are retained in the ER, allowing ER mannosidases to generate Man8-Man5 glycan structures by mannose trimming [5]. It has been proposed that these glycans are the signal for degradation and they are recognized by specific lectin molecules involved in ERAD. EDEM1–3 proteins were traditionally predicted to act as lectins [6], however by sequence similarity these proteins belong to a mannosidase-like group of enzymes known as the Glycosyl hydrolase family 47 (CAZY: GH47, PFAM: PF01532). The mannosidase-like (GH47) domain covers ∼75% of the EDEM1 sequence and due to it, the specificity of EDEM1 for Man8 glycans has been always assumed but never demonstrated. The exact function of the mannosidase-like domain in EDEM1 is also under debate as it is still unclear whether it acts as a mannosidase preparing the substrates for lectin recognition, or it has lost its enzymatic activity and acts solely as a lectin to extract polypeptides from calnexin cycle [7], [8]. Moreover, emerging data suggest that EDEM1 binds to misfolded proteins in a glycan-independent manner [8], [9], point toward the need of identifying new molecular determinants involved in substrate recognition within EDEM1 molecule.

Tyrosinase is constitutively expressed in melanocytes and overexpressed in melanoma cells as a transmembrane glycoprotein with two copper binding domains required for its oxido-reductase activity [10]. This protein is co-and post-translationally folded within the endoplasmic reticulum in the presence of calnexin/calreticulin and is further secreted to the Golgi [11]. A soluble mutant of tyrosinase associates with calreticulin and BiP rather than calnexin and it is retained in the ER and targeted to ubiquitin-proteasomal degradation by ERAD [12]. Incompletely folded transmembrane tyrosinase polypeptides were also reported to be ERAD substrates [13]. Tyrosinase derived peptides are presented at the surface of the melanoma cells and recognized by the infiltrating cytotoxic T lymphocytes [14], [15]. Understanding the degradation pathway of this glycoprotein may be of great interest for the antigen presentation processes. We and others have recently found that EDEM1 associates with seven tyrosinase glycosylation mutants, despite their reduced glycosylation status, possibly being involved in the tyrosinase degradation pathway [16].

Owing to the obscure role of EDEM1 in the degradation of glycosylated proteins, here we investigate its role in the degradation of tyrosinase, a protein whose maturation and processing within the ER are highly dependent on its the N-glycan processing. Overexpression of EDEM1 results in a dramatic reduction in the expression of both wild type and misfolded tyrosinase. We show that aside its mannosidase-like domain, EDEM1 has an intrinsically disordered (ID) region involved in the interaction with tyrosinase mutants, but not with calnexin and SEL1L. Moreover, the absence of the ID region rather than the intact mannosidase-like domain is able to block the accelerated degradation induced by EDEM1 overexpression.

## Results

### EDEM1 modeling reveals the existence of an intrinsically disordered region distinct from the mannosidase-like domain

**Figure 1:**
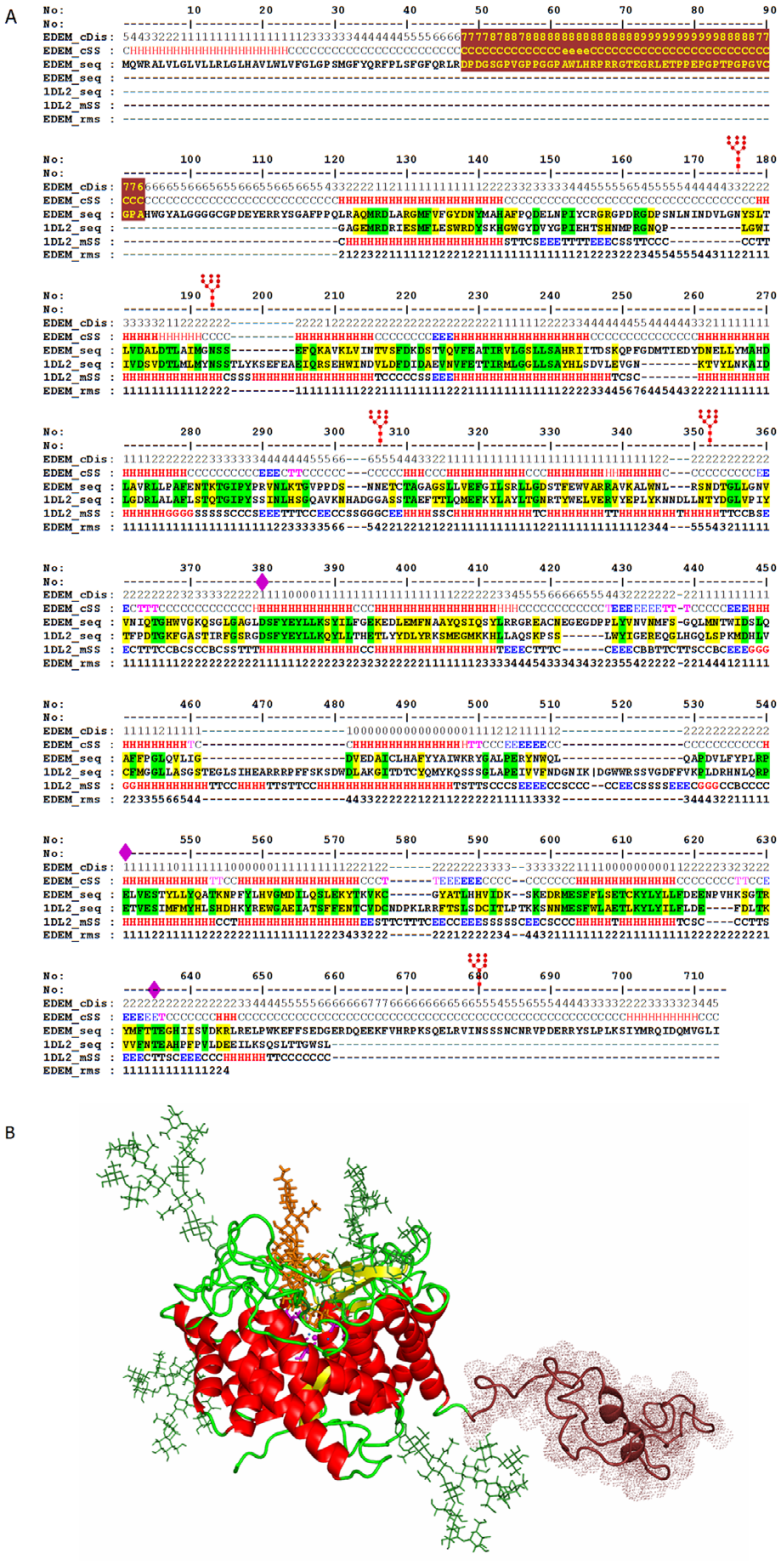
3D model of EDEM1 and sequence alignment to ER class I α1–2 mannosidase template. A. Refined structural alignment of EDEM1 to ER class I α1–2 mannosidase. *EDEM_cDis*; _*cSS* & _*rms* are the *consensus disorder*, *consensus secondary structure* and *model root mean deviation* from an optimal alpha carbon path, in Angstroms, respectively. B. The 3D model of EDEM1. The ID region is shown in dark-red. Helices are shown in red and beta structures in yellow. N-glycans are shown in green lines. A potential Man5 substrate is shown in orange. The calcium ion and the three main aminoacids assessed to be involved in substrate binding and processing are shown in magenta.

Domain recognition methods identify the region aa121–598 (corresponding to aa121–659 in the alignment overlaping frame, O.F., of Fig. 1) of mouse EDEM1 sequence (Genebank: GI_38257724) as a member of the glycosyl transferase 47 family (GH47, PF01532) which includes the ER and Golgi class I α1–2 mannosidases. The top two templates used for modeling were ER class I α1–2 mannosidase from Saccharomyces cerevisiae and Homo sapiens, pdb code 1dl2 and 1×9 d respectively, both with template-target identity of ∼25% and ∼52% sequence similarity. Advanced target to template alignment delineate an overlapping mannosidase like region of 532aa comprising insertions and deletions. This leaves structurally unaccounted two extensions of ∼100 amino acids toward the N-ter end and 54aa toward the C-ter end of the mature protein, after cleavage of the signal sequence. Secondary structure prediction of EDEM1 within the l mannosidase-like domain indicates a very good match with the templates. Locking the functional aminoacids and the secondary structure elements in the overlapping region was further used to refine the alignment and place insertions within the interconnecting loops (Figure 1A). By contrast, the N-ter and C-ter extensions were predicted as mainly coiled, with a short helix in the N-ter signal peptide region. In addition, the N-ter extension shows a high propensity for intrinsic disorder in the region 40 to 119, with highest reaching values between aa48–93. Further analysis showed that this highly disordered region is of low complexity, generally hydrophilic with only few patches of aromatic and hydrophobic motifs, named here the intrinsically disordered (ID) region (Figure 1A, red). The mouse EDEM1 sequence exhibits also five potential N-glycosylation sites in positions N176, N193, N306 (N297 - in the O.F. of Fig. 1), N337 (N352 - in the O.F. of Fig. 1) and N619 (N680 - in the O.F. of Fig. 1). Of these only the first four fall within the mannosidase-like domain and were modeled here.

Based on these sequence propensities we raised a structural model of EDEM1 that comprises the glycosylated mannosidase-like region and one of the many possible random configurations of the N-terminal ID extension of the protein (Figure 1B, dark-red color). In the mannosidase domain region, refinement of inserts aa480–482 (aa534–536 - in the O.F. of Fig. 1) and aa553–556 (614–620 - in the O.F. of Fig. 1) resulted in an increase of the overall model accuracy from 2.63 ? to 2.42 ? root mean square deviation (rmsd), with the global distance test total score raising from GDT_TS = 65.9 to 66.9. The largest local deviation of the model from an optimal alpha carbon path does not exceed 7 ?rmsd and reach its maximum in the insert aa255–259. However, this insert lies far from the mannosidase active site and local structure in the region does not perturb the site configuration, as shown by the Molecular Dynamics stability tests performed on the EDEM1 mannosidase-like domain. In order to identify in EDEM1 model the potential aminoacids involved in glycan recognition and processing, the ligand units located in the active site of two template crystal structures, 1dl2 and 1×9 d, were used to guide a molecular docking of a high mannose GlcNAc2Man9 glycan in the active site of EDEM1 (Video S2).

The binding site of EDEM1 is located in a large cavity formed by the alpha barrel architecture of the GH47 family. Interestingly all the aminoacids forming the inner region of the cavity are very well conserved in all mannosidases including EDEM1. By contrast, the family shows significant sequence variations in loops forming the outer part of the active site cavity that probably secure the specificity for various high-mannose structures.

The conserved architecture of mannosidase binding site imposes the presence of a Ca2+ ion in the inner apex of the site which was shown to bind the terminal mannose (Man -1) of the glycan via O3′ and O4′. The Ca2+ is chelated directly to the protein only via the carbonyl O and Oγ of a threonine - which in EDEM1 is T574 (aa635 - in the O.F. of Fig. 1), and indirectly via 4 structural water molecules [17]. This suggests that in EDEM1, T574 is critical for directing and binding the tip mannose of the ligand.

Trimming of various high mannose structures by various mannosidases is an acido-basic reaction which is carried out by two glutamates that act as a hydrogen donor and acceptor respectively [18]. These residues are conserved in all members of the mannosidase-like family including EDEM1 where they are located in positions E220 and E488 (aa541 - in the O.F. of Fig. 1). On the other hand, studies on human ER mannosidase 1 have shown that of these two aminoacids only mutations at the acceptor result in a 4-fold reduction of substrate affinity [18] suggesting E488 might be also critical for ligand binding in EDEM1.

Furthermore, detailed analysis of glycan-protein contacts in docked EDEM1 model have shown that in addition to T574 and E488, a third aminoacid that might be critical in ligand recognition is D365 (D380 - in the O.F. of Fig. 1, Video S1). This forms two direct hydrogen bonds with the second mannose (Man +1) of the glycan. Studies on human ER mannosidase 1 (Genebank: GI_197304767) have shown indeed that mutations at the equivalent residue D370 completely abolishes the glycan binding to mannosidase [18].

Based on this structural analysis it is therefore expected that a combined triple mutation to D365, T574 and E488 (D380, T635, E541 in the O.F. of Fig. 1) would severely impair the ability of EDEM1 to recognize high-mannose structures.

Based on the model presented in Figure 1 we infer that the ID region might be contributing to the protein-protein interactions of EDEM1, while the mannosidase-like domain might be responsible for the N-glycan recognition and/or processing.

The role of these two domains in EDEM1 functioning is here further investigated.

### Generation of EDEM1 mutants and anti-EDEM1 polyclonal antibodies

**Figure 2:**
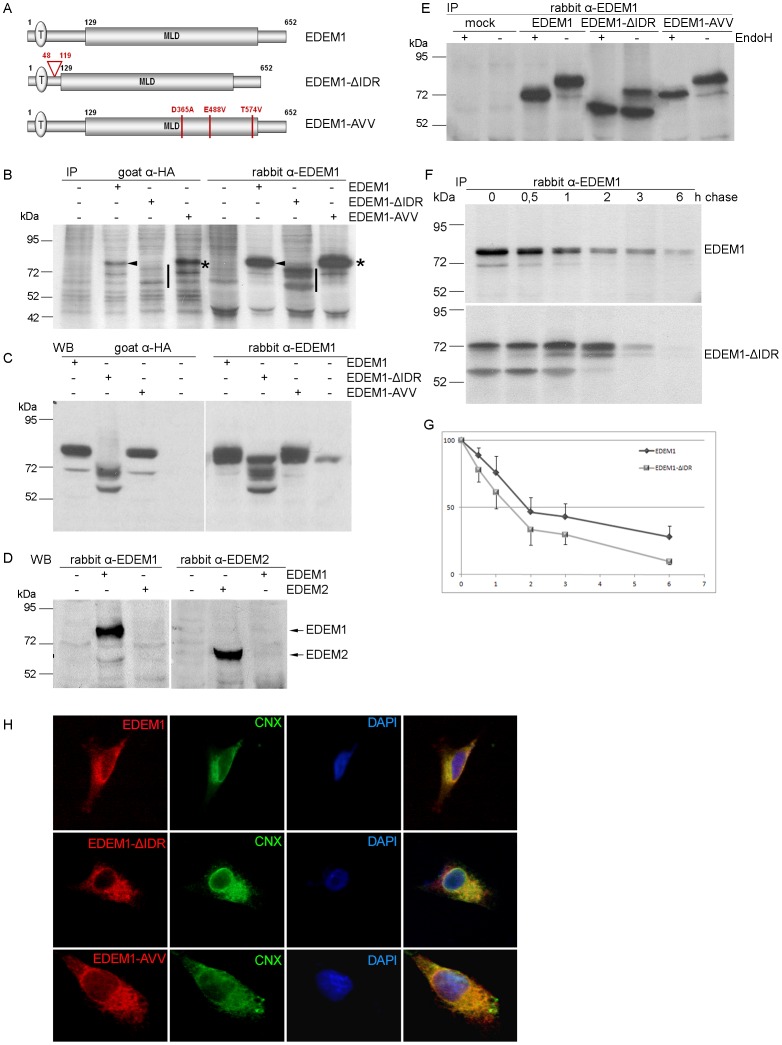
Cloning of EDEM1 mutants and their half-lifes and localization. A. Schematic representation of EDEM1 mutants. The putative transmembrane domain is oval, the mannosidase-like domain (MLD) is, the deletion of the ID region and the point mutants are shown in red. B. EDEM1, EDEM1- ΔIDR and EDEM1-AVV were expressed in HEK 293T cells. The cells were pulse labeled with [^35^S]-Methionine/Cysteine for 20 min and chased for 0 min. Half of the cell lysates were immunoprecipitated with anti-HA antibodies and the other half with anti-EDEM1 antibodies and analyzed by SDS-PAGE followed by autoradiography. Arrowhead indicates EDEM1 band, horizontal line highlights the two bands of EDEM1-ΔIDR and the asterisk points out EDEM1-AVV band. C. 293T cells overexpressing the three EDEM1 proteins grown in 3,5 cm culture dishes were lysed and one tenth of the cell lysate was loaded on gel separated by SDS-PAGE and blotted with goat anti-HA or rabbit anti-EDEM1 antibodies. D. Cells expressing EDEM1 or EDEM2 recombinant constructs were used for western blot experiments, similar to Figure 2C, using rabbit α-EDEM1 or rabbit α-EDEM2 antibodies as mentioned in the figure. E. EDEM1 mutants were transfected in HEK 293T cells, pulse-labeled for 30 min and chased for 0 min. Cell lysates were immunoprecipitated with rabbit anti-EDEM1 antibodies. Samples were divided in two an subjected to EndoH digestion over night and solved by SDS-PAGE. F. To determine the rate of degradation of EDEM1 mutants, HEK 293T cells overexpressing the EDEM mutants were labeled with [^35^S]-Methionine/Cysteine for 20 min and chased for 0 up to 6 h. Total lysates were immunoprecipitated with anti-EDEM1 antiserum and the isolated proteins were separated by SDS-PAGE and visualized by autoradiography. G. The graph shows the average percent EDEM1 remaining after chase relative to corresponding pulse, using Image J software, mean of three independent experiments. H. A375 cells were grown on coverslips for one day, transfected with cDNA encoding for the three EDEM1 mutants and processed for immunofluorescence with polyclonal anti-EDEM1 and rabbit anti-calnexin antibodies.

Two different mutants of EDEM1 were generated and HA-epitope tagged at their C-terminus. The construct EDEM1-AVV is a triple mutant D365A/E488V/T574V and the construct EDEM1-ΔIDR lacks the ID region between amino acids 48–119 (Figure 2A). A polyclonal antiserum was generated against the full length recombinant mouse EDEM1 molecule as described in Materials and methods. The specificity of our antibodies, was compared to previously described antibodies directed to recognize the HA tag attached to EDEM1 [6], [19], [20]. EDEM1 wild type and mutants were expressed, immunoprecipitated from [^35^S]-methionine/cysteine pulsed HEK 293T cells with either anti-HA or anti-EDEM1 polyclonal antibodies and analyzed by SDS-PAGE followed by autoradiography. Anti-EDEM1 polyclonal antibodies recognized EDEM1 proteins with higher specificity than the anti-HA antibodies (Figure 2B). Endogenous EDEM1 was not immunoprecipitated in the non-transfected cells.

Lysates of cells expressing the EDEM1 and wild type and mutants were separated by SDS-PAGE in denaturing conditions and the Western blots probed with goat anti-HA or rabbit anti-EDEM1 antibodies to detect the expression of EDEM1 proteins. Endogenous EDEM1 was detected by Western blot, but was not immunoprecipitated from labeled cells, possibly because of its reported low expression level in HEK 293T cells [21] (Figure 2B,C). Furthermore, the anti-EDEM1 antibodies did not recognize overexpressed EDEM2 used as a control in Figure 2D. These antibodies were further used throughout this study. All exogenously expressed proteins migrated at their expected molecular masses, with EDEM1-ΔIDR migrating as two bands.

To further characterize the EDEM1-ΔIDR mutant, we performed an immunoprecipitation and EndoH digestion experiment in [^35^S]-methionine/cysteine labeled cells which indicated that the two bands detected represent the glycosylated and non-glycosylated form (Figure 2E). Next, we determined the life-span of deletion mutant. Cells overexpressing EDEM1 and the mutant were pulse-chased for the indicated time points and proteins were immunoprecipitated using the antiserum raised against EDEM1. The fastest migrating band of the EDEM1-ΔIDR mutant is a precursor polypeptide that matures into the prominent band during the first two hours of synthesis. As shown in Figure 2F and G, EDEM1 and the deletion mutant show relatively similar half-lifes ranging between 1.5 h (EDEM1-ΔIDR) and 1.9 h (EDEM1), corresponding to the previously reported values for wild type EDEM1 [21].

Exogenously expressed wild type EDEM1 and its two mutants were visualized with anti-EDEM1 antibodies in A375 transfected cells (Figure 2H). They were found within the endoplasmic reticulum by co-localization with the ER resident chaperone, calnexin.

### EDEM1 accelerates the degradation of wild type and misfolded tyrosinase

**Figure 3:**
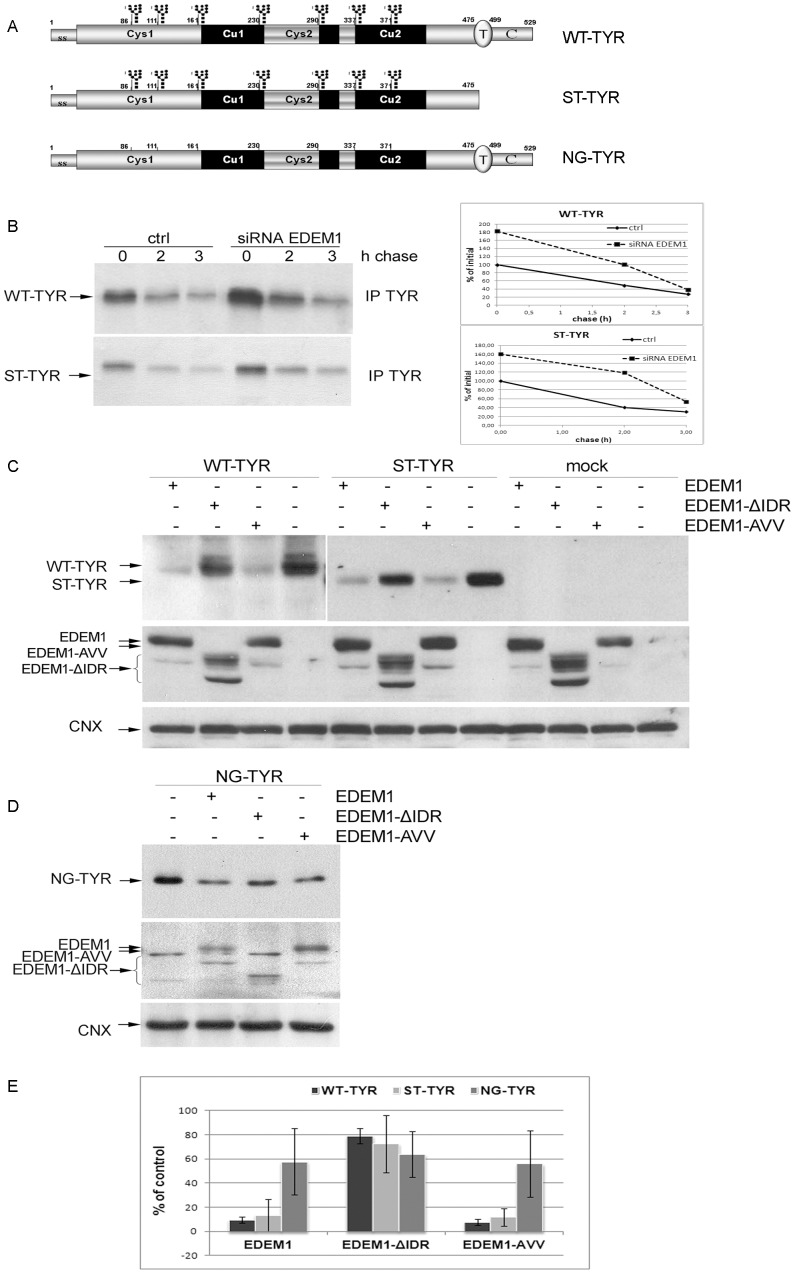
EDEM1 expression modulates the degradation of tyrosinase mutants. A. Schematic representation of tyrosinase mutants; the glycans are shown in black, the transmembrane domain (T) is oval and the C-terminal cytosolic tail (C) is grey. B. HEK 293T cells were co-transfected with plasmid encoding for siRNA for EDEM 1 protein and with WT-TYR or ST-TYR. Cells were pulse-labeled for 20 min, chased for the indicated time points and immunoprecipitated with anti-tyrosinase antibodies. The immunocomplexes were separated by SDS-PAGE and visualized by autoradiography. Graphs show the relative densitometry for the mentioned experiment using ImageJ. C. Tyrosinase wild type and soluble form were co transfected with EDEM1 mutants in 293T cells. One tenth of the cellular lysate was loaded on gel, transferred on membrane and blotted using mouse anti-tyrosinase (T311), goat anti-HA antibodies and goat anti-calnexin antibodies. Calnexin was used as loading control. D. Non-glycosylated tyrosinase was used for the same experiment as in B and cell lysates were used for Western blot with mouse anti-tyrosinase, rabbit anti-EDEM1 and goat anti-calnexin antibodies. E. The graph shows the decrease in tyrosinase expression relative to control sample, average of three independent experiments.

Tyrosinase polypeptide transits the ER and the Golgi on its way to melanosomes, the intracellular site of pigmentation in mammals. Tyrosinase N-glycosylation mutants, as well as truncated tyrosinase lacking its transmembrane domain are genuine ERAD substrates eventually degraded by proteasomes [12], [16], [22]. Here we used wild type, the truncated soluble tyrosinase and the non-glycosylated mutant, represented schematically in figure 3A, as EDEM1 targets.

To investigate the effect of EDEM1 on tyrosinases degradation, 293T cells expressing soluble (ST-TYR) and wild-type tyrosinase (WT-TYR) were transfected with control or EDEM1 siRNA. Cells were [^35^S]-methionine/cysteine pulsed, immunoprecipitated with anti-tyrosinase antibody (T311) and analyzed by SDS-PAGE and autoradiography. Knockdown of EDEM1 increased the expression of soluble tyrosinase by about 50% while delaying its degradation (Figure 3B). Surprisingly, wild type tyrosinase degradation was also delayed, but at lesser extent, all suggesting that not only misfolded, but also wild type tyrosinase may transiently interact with EDEM1.

To investigate further the effect of EDEM1 upon protein degradation, we assessed the degradation of tyrosinases co-expressed with EDEM1 and its two mutants. HEK 293T cells were used to co-transfect tyrosinase and EDEM1 mutants, respectively and the expression of the proteins was detected by Western blot. As seen in figure 3C the degradation of both wild type and soluble tyrosinase was accelerated when co-expressed with native or triple mutant EDEM1 and seemed to be unchanged by the EDEM1 deletion mutant. The middle panel shows the expression of EDEM1 proteins. As a loading control we used calnexin (lower panel). As seen in Figure 3D, similar results were found for non-glycosylated tyrosinase, although the degradation induced by EDEM1 mutants was less pronounced in this case. As the protein is completely misfolded and the effect of EDEM1 overexpression is low, it may require the presence of other molecular mechanisms to provide an efficient degradation. The dramatic reduction in the amount of wild type and soluble tyrosinase upon EDEM1 overexpression was further documented by the quantification of the Western blots shown in Figure 3E.

We determined the half-life of WT-TYR and ST-TYR in the presence of EDEM1 mutants using the cycloheximide inhibition of protein synthesis. HEK293T cells were cotransfected with tyrosinases and EDEM1 mutants as for the above experiments. Afterwards cells were treated with cycloheximide and the level of tyrosinase within the cell was determined up to 4 h of treatement. The WT-TYR level decreased with about half of protein degraded at 1.0 h in the presence of EDEM1, compared to 2.2 h in its absence or in the presence of the deletion mutant. ST-TYR half-life dropped from 1.8 h to 1.00 h upon EDEM1 overexpression and was delayed to 1.8 h in the presence of the deletion mutant. EDEM1-AVV had a similar effect with EDEM1 for both tyrosinases. (No reduction was found in the level of calnexin used as a control (Figure S1))

### Binding of EDEM1 to ERAD substrates requires the intrinsically disordered domain

**Figure 4:**
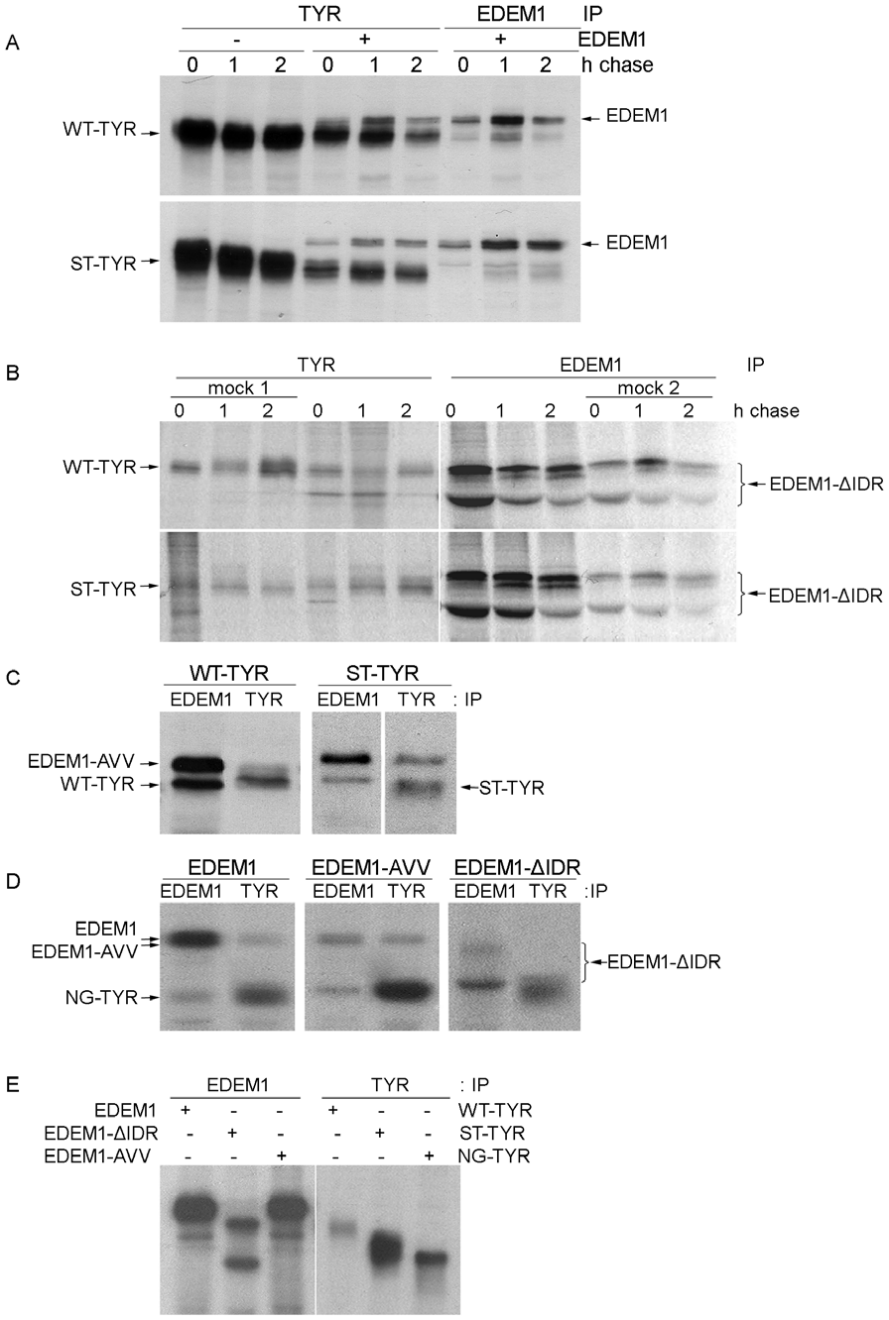
Tyrosinase mutants do not associate with EDEM1 in the absence of the ID region. A. 293T cells were co-transfected tyrosinase mutants, pulse labeled and chased for the indicated time points and the imunocomplexes were isolated using tyrosinase T311 or HA antibodies, separated on gel and visualized by autoradiography. B. The deletion mutant of EDEM1 (EDEM1-ΔIDR) was subjected to the same type of experiment as above, using tyrosinase and EDEM1 polyclonal antibodies. Mock samples were cells transfected with tyrosinase (mock 1) or EDEM1 (mock 2). C. EDEM1-AVV was co-expressed with tyrosinase wild type and soluble mutant and subsequently used for metabolic labeling for 20 min, harvested and immunoprecipitated with tyrosinase or EDEM1 antibodies. D. NG-TYR was co-transfected with EDEM1 mutants in 293T cells which were pulse labeled for 20 min and immunoprecipitated with EDEM1 or tyrosinase antibodies. As shown wild type and triple mutant of EDEM1 coprecipitate with non-glycosylated tyrosinase, but not the truncated EDEM1 mutant(EDEM1-ΔIDR). E. EDEM1 and tyrosinase mutants were expressed in HEK293T cells, pulse labeled 20 min and immunoprecipitated with corresponding antibodies as control for the previously discussed co-precipitations.

To investigate the association of EDEM1 with tyrosinases we established a cellular system co-expressing EDEM1 and WT-TYR and ST-TYR individually. Cells were harvested 24 h post-transfection, pulsed for 20 minutes, chased up to 2 hours and immunoprecipitated with anti-tyrosinase or anti-EDEM1 antibodies. EDEM1 was co-immunoprecipitated with soluble and wild type tyrosinase by anti-tyrosinase antibodies (Figure 4A). Similarly, anti-EDEM1 antibodies co-immunoprecipitated wild type and soluble tyrosinase, although less extensively, possibly because these antibodies may compete for some epitopes engaged in the complex formation.

Since both tyrosinases associated with EDEM1, they were further probed for the interaction with the EDEM1 mutants. Thus, a similar experiment was performed for the deletion mutant, EDEM1-ΔIDR co-expressed with WT-TYR and ST-TYR (Figure 4B, Figure S2). A low amount of the non-glycosylated form of EDEM-ΔIDR was co-precipitated with WT-TYR, while for ST-TYR no significant bands corresponding to the EDEM1 mutant were detected during the chase.

Further, the triple mutant EDEM1-AVV depicted the same pattern as wild type EDEM1, since it was co-precipitated with anti-tyrosinase antibodies in pulse-labeled cells expressing either WT-TYR or ST-TYR as seen in Figure 4C.

As shown in the left panel of Figure 4D, reciprocal co-immunoprecipitation of NG-TYR with EDEM1 was detected in pulse-labeled cells. The same results were obtained when EDEM1 was replaced with the EDEM1-AVV construct (Figure 4D, middle panel). In contrast, the glycosylated form of EDEM1-ΔIDR could not be co-immunoprecipitated from the labeled cells with the NG-TYR mutant (Figure 4D right panel). It should be mentioned that the association with the faster migrating band could not be determined because it overlapped with the NG-TYR mutant. EDEM1 and tyrosinase constructs were specifically immunoprecipitated by anti-EDEM1 and anti-tyrosinase antibodies, respectively, from single-transfected cells (Figure 4E); these controls confirm the specificity of the co-immunoprecipitations in the co-transfected cells.

These data suggest that, without the ID region, EDEM1 fails to associate with the two misfolded tyrosinase mutants. However, even in the absence of the association with EDEM1, the NG-TYR was somehow degraded upon overexpression of the deletion mutant (Figure 3D). Since this is a completely misfolded protein, there may be multiple mechanisms involved in its degradation. Considering the relatively low degradation induced, this maybe within the methods limits, or alternatively, the EDEM1-ΔIDR may indirectly interfere with some of these yet undiscovered degradation pathways. While there was not a non-equivocal answer, it appears that the association with WT-TYR was also weakened. However, the inactivation of the mannosidase-like domain does not impair the recognition by EDEM1.

### EDEM1 associates with calnexin and SEL1L in the absence of the ID region

**Figure 5:**
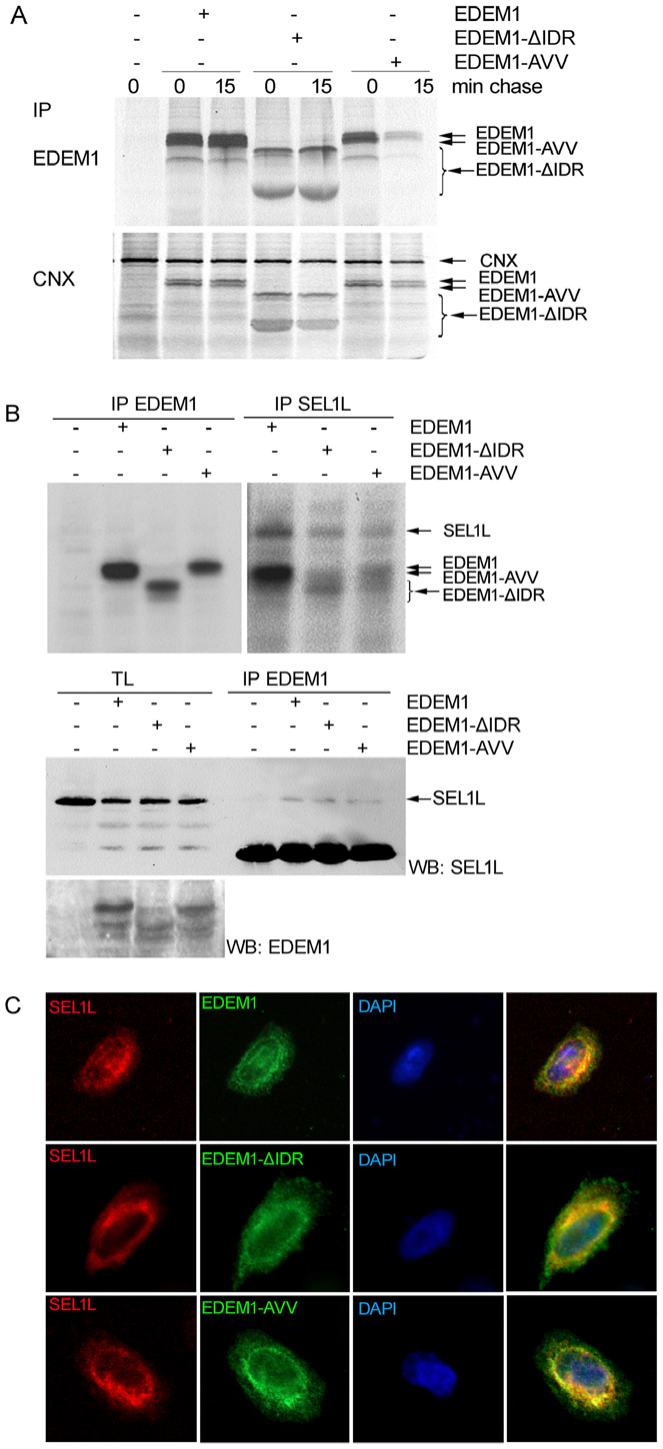
Association of EDEM1 mutants with calnexin and SEL1L. A. HEK 293T cells overexpressing EDEM1 mutants were radioactively labeled for 30 min, chased for 0 and 15 min, harvested and lysed. Cells were immunoprecipitated with anti-EDEM1 or anti-calnexin antibodies and analyzed as in Figure 4. B. (Upper panel). Cells expressing EDEM1 mutants were pulse-labeled for 20 min and chased for 1 h. Half of the cellular lysates were used for immunoprecipitation with anti-SEL1L antibodies and the other half was used for immunoprecipitation with anti-EDEM1 antiserum and analyzed as in A. EDEM1 mutants co-precipitate with SEL1L protein. (Middle panel). Cells expressing EDEM1 mutants were immunoprecipitated with anti-EDEM1 antibodies. The immunocomplexes along with total lysates (TL) controls were separated by SDS-PAGE and Western-blotted with anti- SEL1L antibodies. (Lower panel). Total lysates from the previously described experiment were used for gel electrophoresis and Western blot with EDEM1 antibodies to test the expression of overexpressed mutants. C. A375 cells expressing EDEM1 mutants were processed for immunofluorescence, as described in Experimental procedures, with anti-SEL1L and anti-EDEM1 antibodies.

EDEM1 is able to extract polypeptides that entered the calnexin cycle by direct interaction with calnexin [20]. To test whether the ID region is involved in this interaction, cells were transfected with EDEM1, EDEM1-ΔIDR and EDEM1-AVV, pulsed for 20 min and chased for the indicated times and immunoprecipitated with anti-calnexin antibodies. All EDEM proteins were co-immunoprecipitated with calnexin indicating that indeed the ID region is not required for this interaction (Figure 5A).Controls of immunoprecipitation with cells transfected with a mock plasmid and immunoprecipitated with anti-calnexin antibodies were included, to show the specificity of the co-immunoprecipitations (Figure 5A).

EDEM1 and the proteins with mannose phosphate receptor homology domains, such as Yos9/OS9 and XTP3B [23], [24] are able to deliver the substrate to SEL1L (an ortholog of the yeast Hrd3p) which is involved in the protein dislocation from the ER [25]. Prior reports indicated that EDEM1 requires a functional mannosidase domain for interaction with SEL1L [9]. To determine the molecular determinants of this interaction, cell lysates of pulse-chased cells were divided in two and immunoprecipitated using either anti-EDEM1 control or anti-SEL1L antibodies. As shown in figure 5B upper panel, the anti-SEL1L antibodies co-immunoprecipitated EDEM1 wild type and the mutants.

The pulse-chase experiments were further confirmed by an experiment of immunoprecipitation and Western blot when the natively formed complexes were immunoprecipitated with anti-EDEM1 antibodies, loaded on gel and blotted with anti-SEL1L antibodies. As controls for the anti-SEL1L antibodies, a tenth of the cell lysates (TL) were run on the same gel (Figure 5B, middle panel). Another tenth of the TL, used as a control for the expression of EDEM1 mutants, was separated by SDS-PAGE and blotted with anti-EDEM1 antibodies (Figure 5B lower panel). SEL1L was detected in the isolated complexes of EDEM1, EDEM1-ΔIDR and EDEM1-AVV (Figure 5B, middle panel).

As found for the chaperone calnexin, SEL1L associates with EDEM1 independently of the ID region domain. The interaction could occur within the mannosidase-like domain, but it appears that the domain does not necessarily require its mannosidase-like activity.

Through the use of immunofluorescence, we confirmed co-localization of overexpressed EDEM1 proteins with SEL1L. Partial co-localizations of EDEM1 proteins with SEL1L were visualized with EDEM1 proteins showing a wider ER distribution than SEL1L which was more restrained in some ER domains (Figure 5C).

## Discussion

The results presented in this study indicate that EDEM1 has an intrinsically disordered region required for the co-immunoprecipitation of tyrosinase, an ER transient protein with low folding efficiency and of its misfolded mutants destined to cytosol degradation.

We generated a homology model on the crystal structures of human and Saccharomyces cerevisiae ER class I α1,2-mannosidases and focused on the role of the major domains in EDEM1 recognition step. The major mannosidase-like domain located between aminoacids 121–598 fits with high accuracy an alpha barrel structure able to bind high mannose glycans. In contrast with this highly ordered domain, we have identified an N-terminal region located between aminoacids 40–119, predicted to be intrinsically disordered. Disordered regions can adopt a vast ensemble of unfolded configurations as those seen in denatured proteins [26], [27], [28]. This confers to ID regions the necessary conformational plasticity that enables them molding to fit the binding surfaces of partners in protein-protein interactions [29]. It is well recognized now that due to this property ID regions are present in many eukarial hub proteins that mediate multiple recognition or co-ordinate regulatory events [30], [31]. The presence of ID regions in such protein hubs allow short stretches of interaction prone motifs, named MoRFs, to expose, scaffold and interact with numerous proteins [32].

To determine whether the ID region or the mannosidase-like domain/glycan binding domain of EDEM1 is involved in its recognition step, we also used tyrosinase, a glycoprotein that folds with reduced efficiency into its native conformation. Tyrosinase folding is further decreased upon gene mutations, with some mutants reported to be ERAD substrates in oculocutaneous albinism. In this study we found that wild type tyrosinase, its soluble albino mutant and the non-glycosylated tyrosinase mutant co-immunoprecipitated with EDEM1 independent of their glycosylation state. In addition, the binding was maintained when the carbohydrate binding domain was highly impaired in the triple EDEM1 mutant, indicating that the mannosidase-like domain may not be crucial in the substrate recognition step. Similarly, the α1-antitrypsin mutant bound a different EDEM1 triple mutant within the mannosidase-like domain and the folding-defective BACE 457 co-precipitated the E220Q mutant [9], [33]. More recently, an EDEM1 deletion mutant lacking a fragment of the mannosidase-like domain was reported to enhance the ERAD of the asialoglycoprotein receptor precursor [34]. Thus, EDEM1 binds tyrosinase and its mutants independent of glycans, in a very similar way with the previously reported EDEM1 substrates, indicating that EDEM1 can interact with the polypeptide and requires more than a carbohydrate binding region for the recognition step.

Our data strongly suggest that the ID region is another domain crucial for EDEM1 binding. In the absence of the ID region, this interaction was abolished for the two misfolded mutants, and was very weak for wild type tyrosinase. In the case of wild type tyrosinase, it is quite surprising, since EDEM1 interaction with wild type proteins was not characterized before. However, we also detected a stabilization of wild type tyrosinase similar with that of the truncated tyrosinase upon EDEM1 down regulation. One possibility is that EDEM1 recognizes the incompletely folded molecules of wild type tyrosinase, that are not export competent but rather retained in the ER. This is consistent with previously published reports showing that a substantial amount of the newly synthesized tyrosinase is retained in the ER and degraded by proteasomes [12], [35]. Moreover, wild type tyrosinase is retained in the ER and degraded by ERAD in amelanotic melanoma [36].

A possible scenario is that once bound by the ID region of EDEM1, the glycosylated and nonglycosylated misfolded or immature proteins are recruited to the degradative pathway. This could occur independently of an intact glycan binding domain and possibly independent of the mannose trimming of the substrate. In this scenario, EDEM1 could act as folding sensor of the ERAD substrates. It is tempting to speculate that the ID region of EDEM1 has a unique ability to sense and deliver misfolded proteins independent of their glycosylation status downstream to the ERAD pathway. This would imply that misfolded glycoproteins released from the calnexin/calreticulin cycle and misfolded non-glycosylated polypeptides are all sent to degradation by EDEM1. However, future studies involving a palette of proteins that mature within the ER should be performed to confirm this hypothesis.

The efficiency of EDEM1 in accelerating the ERAD presumably comes for its ability to further deliver the substrates to the SEL1L complex. SEL1L is a membrane adaptor protein involved in the ubiquitinating complex associated with the ER membrane that directly delivers the non-native proteins to the proteasomal degradation. An interaction between SEL1L and EDEM1 has been previously reported [9]. We found that the ID region is solely specific for the ERAD substrates and its deletion does not impair the SEL1L association. The mannosidase-like domain is however required for the SEL1L interaction, although from our studies its intact function appears to be unnecessary. This association is reduced, but not abolished, in the presence of kifunensine [9]. Therefore the mechanism of this inhibition is not clear, since kifunensine may inhibit either the SEL1L mannose trimming, or may block the EDEM1 active center. Nevertheless, we propose that the binding of EDEM1 with SEL1L could be more complex than previously thought, since it may take place even in the absence of the ID region or the intact mannosidase-like domain.

We propose a model where EDEM1 binds the ER transit proteins using also the N-terminal ID region. Whether the binding is direct or it implies another component of the ERAD pathway, it remains to be further investigated. At least in the case of tyrosinase, the N-glycosylation process was not required for the binding of EDEM1. Association of EDEM1 with calnexin and SEL1L occurs independently of the ID region, presumably through other domains of the EDEM1. Identification of other ERAD substrates dependent on this mechanism and the understanding of EDEM1 regulation under ER stress conditions, will help in understanding the specialized ER processing mechanisms adapted to maintain the protein homeostasis in non-pathological state.

Finally, tyrosinase is a tumour antigen able to generate peptides that are presented at the cell surface in association with MHC-I. Overexpression of EDEM1 in melanoma cells could be an efficient way of accelerating tyrosinase degradation and inducing a more efficient display of peptides at the surface of the melanoma cell leading to an efficient recognition by CTL and the eventual enhanced elimination of melanoma cells. Whether EDEM1 may play an active role in the enhanced antigen presentation of tyrosinase peptides in melanoma cells is currently under investigation.

## Materials and Methods

### Sequence analysis and modeling

The EDEM1 sequence was assessed for patterns, motives and domain delineation in with InterPro [37]. The sequence was profiled for various structural propensities such as: secondary structure, intrinsic disorder, contact formation, accessibility etc. Intrinsic disorder was assessed with a consensus score raised from DisEMBL [38], RONN [39], IUPRED [40]. Consensus secondary structure propensity was determined starting from SSPro [41], GOR IV, SOPMA and HNN [42]. Template identification was performed with Phyre [43]. Refined alignment and sequence to structure mapping in the target-template overlapping region were performed in Insight II and with Slide, an interactive threading software [44].

Model building was performed with the Homology module in Insight II software from Accelrys. Local refinement consisted in iterative variable loop rebuilding followed by energy optimization and model quality assessment using MetaMQAP [45]. Global optimization consisted in repeated rounds of energy minimization, and local and global simulated annealing performed with Discover in Insight II using the cvff force field. The glycan moiety was modeled with Glyco-Pack, a software for glycoprotein structural analysis and modeling [46] and with Amber [47]. Four GlcNAc2Man9 N-glycans were attached in silico to the EDEM1 model at glycosylation sites s1∶N176, s2∶N193, s3∶N294 and s4∶N340 in configurations consistent with the most populated conformer derived from the SAGS data base [48]. The glycan attachment, conformational search, and clash analysis were performed with Glyco-Pack and the glycoprotein structure was further optimized in Amber 10, using Glycam 06 and ff99sb force fields [49]. Stability tests consisted in Molecular Dynamics runs performed at constant temperature and pressure with PME summation over periodic boundary conditions. The trajectories were generated with NAMD 2.7b1 [50] on a Bull NovaScale HPC cluster with 32 CPU cores. The water box solvated model of EDEM1 mannosidase-like domain was built using CHARMm 22 force field with CMAP corrections and the net electric charge was neutralized using the meadionize VMD plugin. The VMD suite [51] and custom scripts were used to analyze the MD trajectories.

### Reagents, cell culture, antibodies

HEK 293T (human embryonic kidney cells) and A375 (amelanotic melanoma) cells (European Collection of Animal Cell Cultures, Porton Down, UK), were cultivated in DMEM medium (Biochrom, Cambridge, UK) supplemented with 10% fetal calf serum (Biochrom, Cambridge, UK), 50 units/ml penicillin, 50 mg/ml streptomycin (Gibco, Paisley, UK), 20 mM glutamine (Gibco, Paisley, UK) and maintained at 37°C with 5% CO2. Mouse anti-tyrosinase (T311) antibody (sc-20035), goat anti-SEL1L (sc-48081), goat anti-HA antisera (sc-805-G), were from Santa Cruz Biotechnology, Heidelberg, Germany. Rabbit anti-calnexin (Adi-SPA-865-F) antibodies were from Stressgen. Rabbit polyclonal anti-EDEM 1 antibodies were produced in our laboratory: the cDNA corresponding to full length mouse EDEM1 was cloned into pHAT-2 (Clontech, Saint-Germain-en-Laye, France) bacterial expression vector with 6His tag. The proteins were expressed in E. coli BL 21RIL bacterial strains (Novagen Darmstadt, Germany) and affinity purified from bacterial cultures. Purified proteins were injected to rabbit along with Freud adjuvant (Sigma, St. Louis, US) and after five boosts, the antiserum was collected. EndoH (New England Biolabs, Herts, UK), protein A-Sepharose and protein G-Sepharose from Invitrogen cycloheximide was from Sigma-Aldrich. Si-RNA experiments were performed using siEDEMpcDNA3, a kind gift of Dr. M. Molinari [6].

### Plasmids

Wild type (WT) cDNA of mouse EDEM1 cloned into pCMV-Sport was a kind gift from Dr. K. Nagata and Dr. N. Hosokawa (Institute of Frontier Medical Science, Kyoto University) [19]. The EDEM1-ΔIDR mutant that lacks the aminoacids 48–119 was constructed by three-step PCR method using the following mutagenic set of primers: 5′-CCTCAAGCGCTGGAAGCCGAAGC and 5′-GCTTCGGCTTCCAGCGCTTGAGGCAGCTGCGTGCCCAGATGCGCGAC. Point mutations at D365A, E488V, and T574V were introduced by three-step PCR with corresponding mutagenic primers. The EDEM1-AVV was obtained by PCR with the mutagenic primers corresponding to E488V mutation, based on the cDNA sequences of D365A and T574V mutants. The non-glycosylated tyrosinase NG-TYR was created by three-step PCR method with mutagenic primers corresponding to s4-N230, based on the sequence of TYR-Δ1,2,3 and TYR-Δ5,6,7 previously described [16]. All DNA constructs were sequence verified.

### Cell transfection

Cells were co-transfected with pTriEx-TYR and pCMV SPORT2-EDEM1 using polyethylene-imine (PEI) (Sigma) for HEK 293T and Lipofectamine 2000 (Invitrogen) for A375 cells. The transfection mix was prepared in serum free media for cells plated on 3.5-cm dishes. Cells were harvested 24 h or 48 h after transfection and lysed in HEPES-CHAPS lysis buffer (2% CHAPS in 50 mM Hepes, 200 mM NaCl, pH 7.5) (Sigma), containing a mixture of protease inhibitors (Roche), for 30 min, on ice.

### Pulse-chase analysis

The cells were starved in the cysteine/methionine-free medium for 30 min, pulse labeled for 20 or 30 min with 50–75 mCi of [^35^S]-methionine/cysteine (Tran35S-label, 1100 Ci/mmol, MP Biomedicals) and chased for the indicated time points. Labeled cells were washed with cold PBS and lysed with CHAPS lysis buffer, as mentioned above.

### Immunoprecipitation

Protein A/G-sepharose beads, antisera and cell lysates were incubated for 24 h at 4°C. Immunoprecipitates were washed 4 times with wash buffer, (0.5% CHAPS in HEPES buffer) resuspended in TE-buffer (10 mM Tris-HCl, pH 6.8, 1 mM EDTA) with DTT and heated to 95°C for 5 min in Laemmli sample buffer (200 mM Tris-Cl pH 6.8, 3% SDS, 10% glycerol, 1 mM EDTA and 0.004% bromophenol blue, final concentrations). The samples were analyzed by SDS-PAGE and proteins were visualized by autoradiography.

### Immunoprecipitation and Western blot

Transfected cells were harvested, lysed and subjected to immunoprecipitation with antibodies against EDEM1, samples were loaded on 10% SDS-PAGE gel and subsequently used for Western blot with SEL1L. Results are visualized by chemiluminescent reaction.

### EndoH digestion

Immunoprecipitated [^35^S]-labeled samples were eluted from protein A-Sepharose slurry in SDS 1% by incubation for 5 min at 100°C. The samples were cooled and mixed with 1/10 EndoH reaction buffer (0.5 M sodium citrate, pH 5.5). 250 units of EndoH (0.5 µl) was added to one-half of the amount of each sample, whereas the other half contained EndoH buffers alone. The digested samples, together with non-digested controls, were incubated for 18 h at 37°C.

### Western blot

HEK293T cells previously transfected were lysed and one tenth of total lysates were loaded on gel for each sample. Proteins separated by SDS-PAGE were afterwards transferred on nitrocellulose membrane and probed with anti-tyrosinase (T311), anti-EDEM1 or anti-calnexin antibodies, for 1 hour at room temperature. For chase we used 50 µM final concentration and cells were incubated for 0, 1, 3 and 4 h with media containing cycloheximide. The Western blot detection was performed as stated above.

### Fluorescence microscopy

A375 cells were seeded onto glass coverslips and allowed to attach overnight and next cells were transfected as stated above. Fixation was achieved with 4% paraformaldehyde for 20 min at room temperature. Cells were permeabilized with 0.1% Triton X-100 for 3 min at room temperature and incubated with antibodies for the proteins of interest and subsequently with corresponding AlexaFluor conjugated secondary antibodies. Imaging was performed on Nikon Eclipse E 600 fluorescent microscope.

## Supporting Information

Video S1 **Top view of the EDEM1 model showing the location of the three point mutations.**
(AVI)

Video S2 **EDEM1 Lateral view of the EDEM1 model with Man9 docked in the active site of the mannosidase-like domain.**
(AVI)

Figure S1 **Tyrosinase half-life detemined by cycloheximide chase.** A. HEK 293T cells co-transfected with tyrosinase mutants and empty vector were incubated with cycloheximide for the indicated time points. Cells were harvested and one tenth of total lysates was loaded on gel, transferred on nitrocellulose membrane and blotted for tyrosinase, EDEM1 and calnexin. B. The same experiment as above was made for EDEM1 wild type protein cotransfected with tyrosinase mutants. C. EDEM1-ΔIDR was co-transfected with tyrosinase mutants and used for the same experiment. D. EDEM1-AVV was subjected to the same type of experiment to determine the turnover of tyrosinase proteins. E. The Western blots from previous experiments were quantified using ImageJ software and the results are depicted here for each of tyrosinase mutants.
(TIF)

Figure S2 **A. Schematic representation of tyrosinase mutant Δ567-TYR.** B. Cells co-transfected with tyrosinase mutatns and EDEM1-ΔIDR were pulse labeled for 30 minutes and used for immunoprecipitation either with tyrosinase antibodies or EDEM1 polyclonal antibodies. No co-immunoprecipitation was detected for any of the samples used for experiments. C. HEK293T cells co-transfected with tyrosinase Δ567 mutant and EDEM1 were pulse-labeled for 30 minutes and immunoprecipitated with tyrosinase or EDEM1 antibodies and visualized by autoradiography.
(TIF)
