# Supplementary figures and images for: Tyrosinase Degradation Is Prevented when EDEM1 Lacks the Intrinsically Disordered Region
Source: PLoS One. 2012 Aug 8;7(8):e42998. doi: 10.1371/journal.pone.0042998 (PMC3414498; doi:10.1371/journal.pone.0042998)

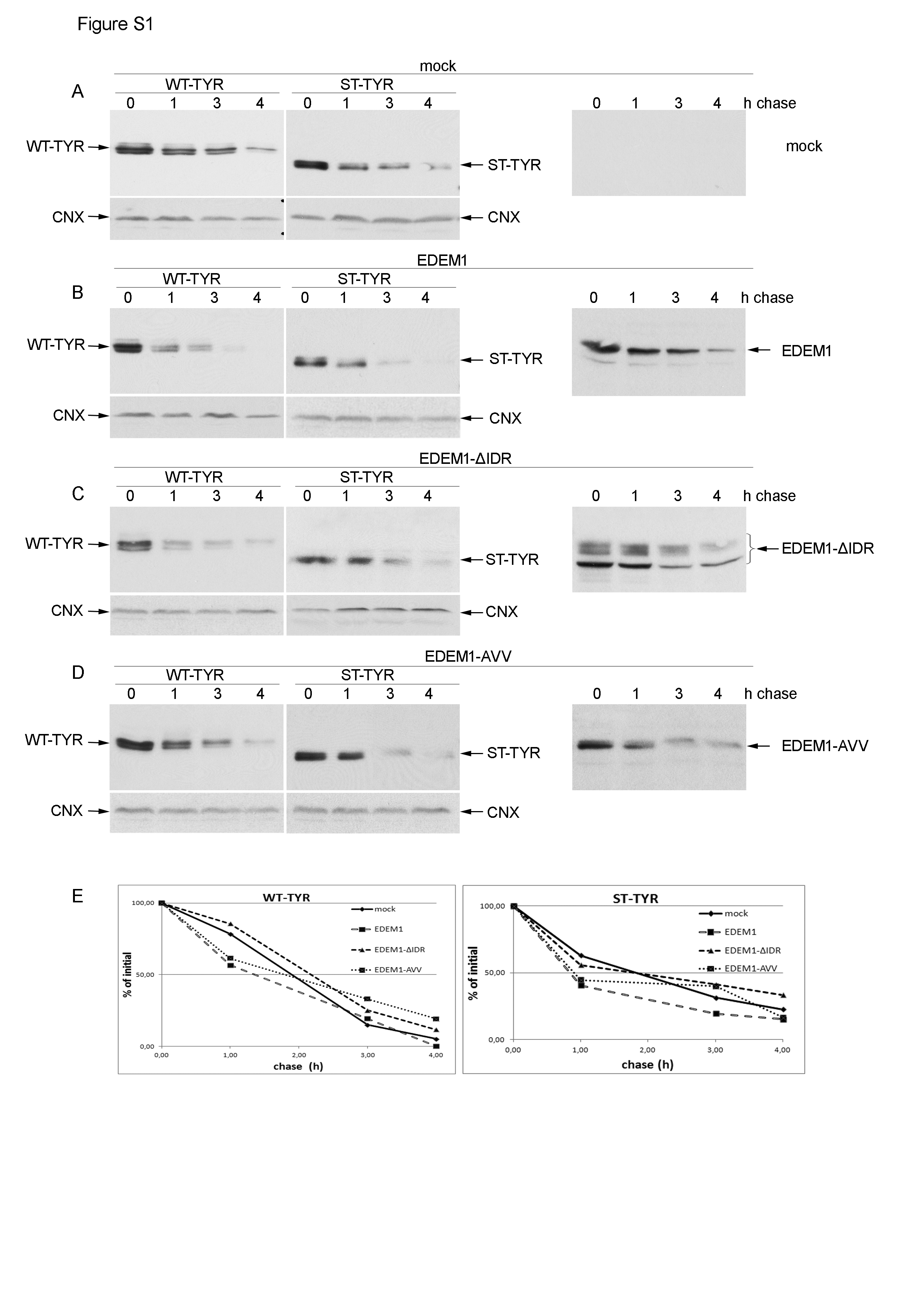

Supplement: Figure S1 — Tyrosinase half-life detemined by cycloheximide chase. A. HEK 293T cells co-transfected with tyrosinase mutants and empty vector were incubated with cycloheximide for the indicated time points. Cells were harvested and one tenth of total lysates was loaded on gel, transferred on nitrocellulose membrane and blotted for tyrosinase, EDEM1 and calnexin. B. The same experiment as above was made for EDEM1 wild type protein cotransfected with tyrosinase mutants. C. EDEM1-ΔIDR was co-transfected with tyrosinase mutants and used for the same experiment. D. EDEM1-AVV was subjected to the same type of experiment to determine the turnover of tyrosinase proteins. E. The Western blots from previous experiments were quantified using ImageJ software and the results are depicted here for each of tyrosinase mutants. (TIF) [file pone.0042998.s003.tif]

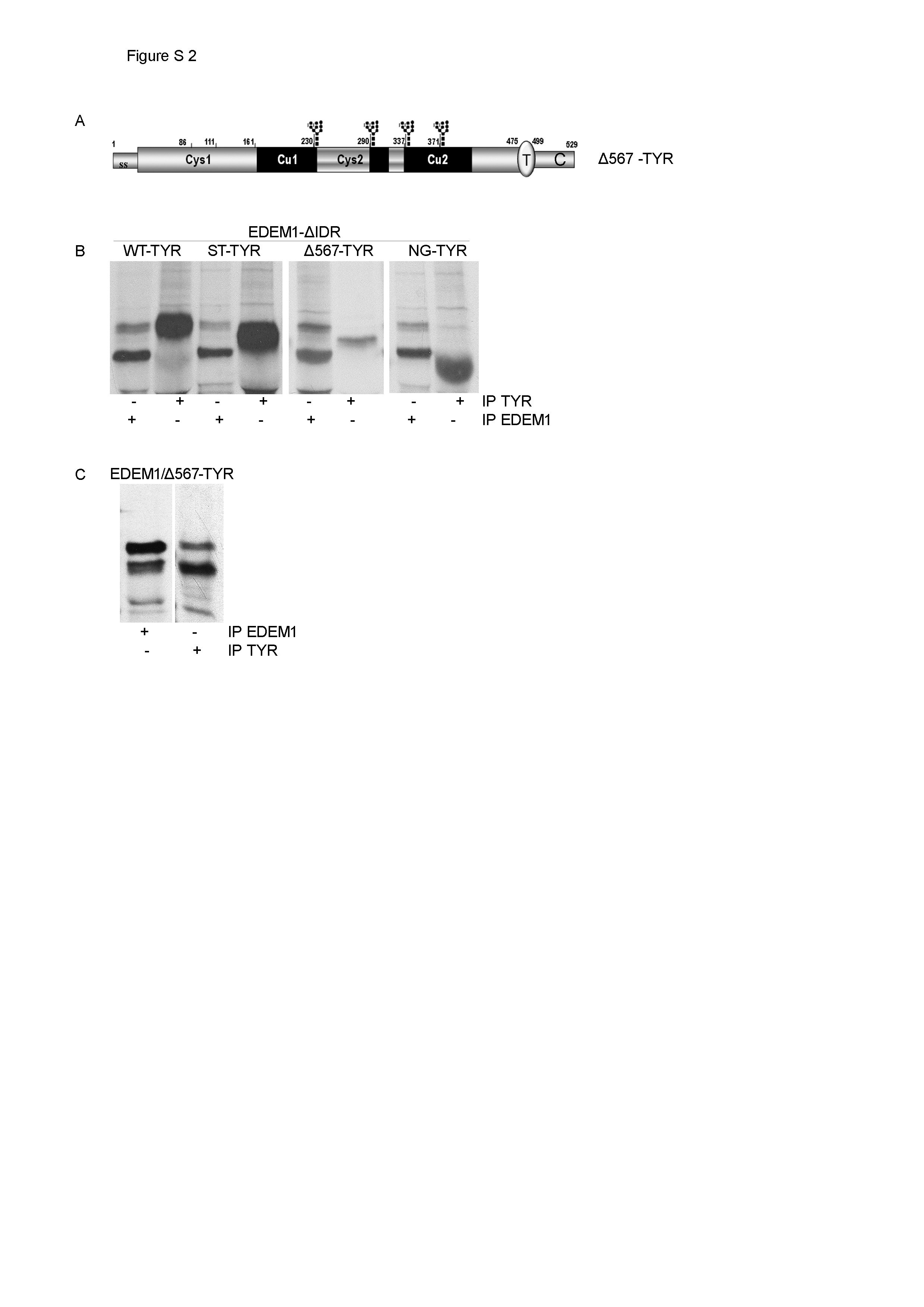

Supplement: Figure S2 — A. Schematic representation of tyrosinase mutant Δ567-TYR. B. Cells co-transfected with tyrosinase mutatns and EDEM1-ΔIDR were pulse labeled for 30 minutes and used for immunoprecipitation either with tyrosinase antibodies or EDEM1 polyclonal antibodies. No co-immunoprecipitation was detected for any of the samples used for experiments. C. HEK293T cells co-transfected with tyrosinase Δ567 mutant and EDEM1 were pulse-labeled for 30 minutes and immunoprecipitated with tyrosinase or EDEM1 antibodies and visualized by autoradiography. (TIF) [file pone.0042998.s004.tif]
